# ﻿Cave-inhabiting Cheliferidae (Arachnida, Pseudoscorpiones) from Thailand, with description of four new species of *Metachelifer* Redikorzev

**DOI:** 10.3897/zookeys.1103.78808

**Published:** 2022-06-03

**Authors:** Yun-Chun Li, Ai-Min Shi

**Affiliations:** 1 College of Life Science, China West Normal University, Nanchong, Sichuan 637009, China China West Normal University Nanchong China; 2 Key Laboratory of Southwest China Wildlife Resources Conservation, Institute of Rare Animals & Plants, China West Normal University, Nanchong, Sichuan 637009, China China West Normal University Nanchong China

**Keywords:** Identification key, pseudoscorpion, Southeast Asia, taxonomy, troglobiont

## Abstract

Four new species of the genus *Metachelifer* Redikorzev, 1938 are described from caves in the provinces of Tak (*M.takensis***sp. nov.** and *M.thailandicus***sp. nov.**), Chiangmai (*M.mahnerti***sp. nov.**), and Nakhon Ratchasima (*M.cheni***sp. nov.**). An identification key is provided to all known world representatives of the genus *Metachelifer*.

## ﻿Introduction

The pseudoscorpion genus *Metachelifer* Redikorzev, 1938 belongs to the family Cheliferidae Risso, 1827 and the subfamily Cheliferinae. This subfamily contains 57 genera that are mostly distributed in Africa, southern Europe, central Asia, North America, and South America (World Pseudoscorpiones Catalog 2022). At present, *Metachelifer* contains three species which are confined to Asia: *M.duboscqui* Redikorzev, 1938 from Cambodia, Laos, Philippines, and Vietnam; *M.macrotuberculatus* (Krumpál, 1987) and *M.nepalensis* (Beier, 1974) from Nepal (Redikorzev 1938; Beier 1974; Krumpál 1987).

Males of the genus *Metachelifer* can be characterized by the carapace surface with tubercles; coxa IV with an anterolateral process and coxal sac; sternite III uplifted laterally and extending to short thorns; leg I tarsus lateral claw shorter than mesal one; subterminal seta simple (Redikorzev 1938; Dashdamirov 2006). While identifying pseudoscorpion specimens collected from Thailand in 2014–2016, four new cave-inhabiting species of *Metachelifer* were found and are described here.

## ﻿Materials and methods

The specimens were examined with a Leica M205FA stereomicroscope and an Olympus CX31 compound microscope. The specimens are preserved in 75% ethanol. They were cleared in lactic acid for 12–24 h at room temperature and, after the study, washed in distilled water and returned to alcohol. Photographs were taken using a Canon 6D Mark II camera fitted with a Laowa 25 mm f/2.8 2.5–5 × and 100 mm F2.8 2.0 × ultra macro lens. The final high depth of field images were stacked from 30 to 80 single photos using Helicon Focus 7.6.1., and CorelDRAW 2018 and SAI 2 software were used to draw the figures. The type specimens of the new species are deposited in the collection of the Museum of China West Normal University (**MCWNU**; Sichuan, China) and Muséum national d’Histoire naturelle (**MNHN**; Paris, France).

Pseudoscorpion terminology and measurements mostly follow Chamberlin (1931) with some minor modifications to the terminology of the trichobothria (Harvey 1992) and chelicera (Judson 2007). The following abbreviations are used for the trichobothria: *b* = basal; *sb* = sub-basal; *st* = sub-terminal; *t* = terminal; *ib* = interior basal; *isb* = interior sub-basal; *ist* = interior sub-terminal; *it* = interior terminal; *eb* = exterior basal; *esb* = exterior sub-basal; *est* = exterior sub-terminal; *et* = exterior terminal.

## ﻿Results

**Cheliferidae Risso, 1827**

### Cheliferinae Risso, 1827

#### ﻿ Metachelifer

Taxon classificationAnimaliaPseudoscorpionesCheliferidae

Redikorzev, 1938

77077E5B-0AE8-514D-ACFA-38A4C3A10EF4

Metachelifer Redikorzev, 1938: 108.

##### Type species.

*Metacheliferduboscqui* Redikorzev, 1938, by monotypy.

#### ﻿Identification key to the species of *Metachelifer*

**Table:** 

1	Carapace slightly broader than long	**2**
–	Carapace slightly longer than broad	**3**
2	Tergite XI with two tactile setae; pedipalpal femur 4.49 × longer than broad (1.08/0.24 mm)	***M.macrotuberculatus* (Krumpál, 1987)**
–	Tergite XI without tactile setae; pedipalpal femur 5.22 × longer than broad (1.41/0.27 mm)	***M.nepalensis* (Beier, 1974)**
3	Fixed and movable chelal fingers with at least 57 teeth; trichobothrium *st* distinctly closer to *sb* than to *t*	**4**
–	Fixed and movable chelal fingers with 35 teeth; trichobothrium *st* midway between *sb* and *t*	***M.duboscqui* Redikorzev, 1938**
4	Tergite XI with two tactile setae	**5**
–	Tergite XI without tactile setae	***M.mahnerti* Li & Shi, sp. nov.**
5	Venom ducts very short, not extending past *et* (Figs 1G, 4F); posterior genital operculum of female without lyrifissures	**6**
–	Venom ducts long, extending past *et* (Fig. 3G); posterior genital operculum of female with eight lyrifissures	***M.takensis* Li & Shi, sp. nov.**
6	Carapace with 100–101 setae; coxal sac occupying 1/2 of coxal length; anterior genital operculum of female with tubular setae	***M.cheni* Li & Shi, sp. nov.**
–	Carapace with 86–88 setae; coxal sac occupying only 2/5 of coxal length; anterior genital operculum of female without tubular setae	***M.thailandicus* Li & Shi, sp. nov.**

#### ﻿ Metachelifer cheni sp. nov.

Taxon classificationAnimaliaPseudoscorpionesCheliferidae

09CC55CD-24C0-5AC0-A2D7-25A1FC04D01D

http://zoobank.org/06F1B0AB-CAE5-461F-A445-53C976611A5C

Figs 1 , 5A, B

##### Type material.

***Holotype*** male: Thailand, Nakhon Ratchasima Province, Pak Chong district, Musee Village, Wat Dewaroop Song Cave 3, 14°33.714'N, 101°24.049'E, 402 m a.s.l., 24 Oct. 2014, Yun-Chun Li and Zhi-Gang Chen leg., in MCWNU (Ms20141014-01). ***Paratypes***: 3 males, 7 females, 7 tritonymphs, collected with the holotype, in MCWNU (Ms20141014-01); 1 male, 2 females, 1 tritonymph, collected with the holotype, in MNHN.

##### Diagnosis.

Troglobiont habitus. This new species is distinguished from other members of the genus *Metachelifer* by the following combination of characters: carapace with 100–101 setae; coxal sac occupying 1/2 of coxal length; male anterior genital operculum without tubular setae; female anterior genital operculum with 31 setae (24 of them tubular) and two lyrifissures; posterior operculum with 12 setae, without lyrifissures.

##### Etymology.

Latinized adjective, derived from the last name of the collector, Zhi-Gang Chen.

##### Description.

**Adult male** (Fig. 5A). Carapace, pedipalps and tergites I–III brown, remaining parts yellowish brown (Fig. 5A).

**Figure 1.**
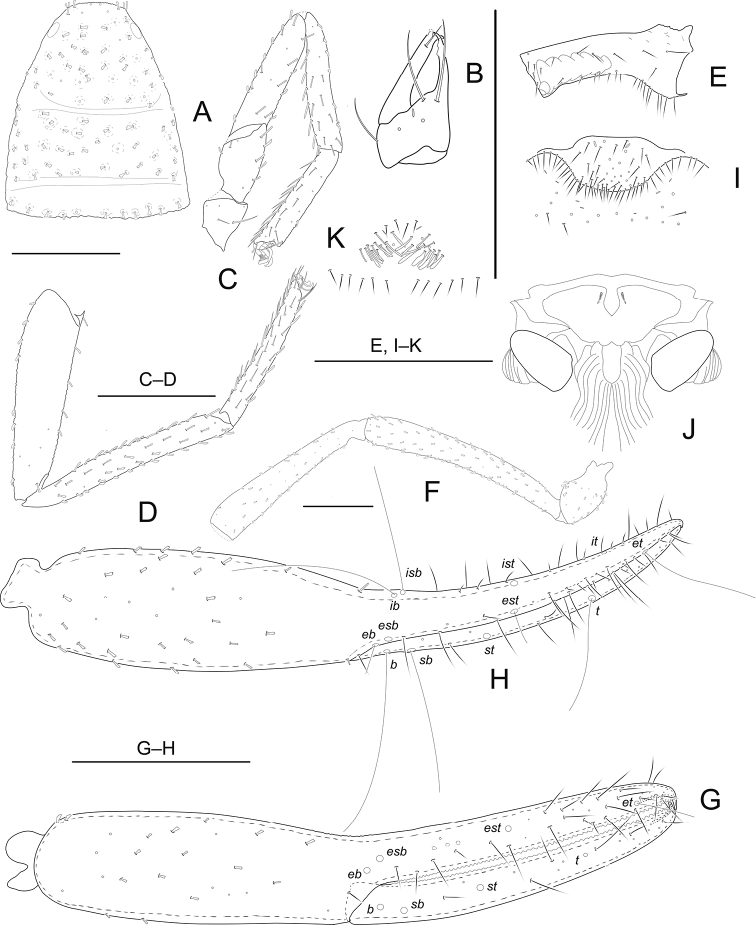
*Metachelifercheni* sp. nov., holotype male (**A–J**) and paratype female (**K**) **A** carapace **B** left chelicera **C** right leg I, lateral view **D** right leg IV, lateral view **E** coxa IV, ventral view **F** palp (minus chela) **G** chela, retrolateral view **H** chela, dorsal view **I** male genital area **J** male genitalia, dorsal view **K** female genital area. Scale bars: 0.50 mm.

***Carapace*** (Fig. 1A): 1.14–1.16 × longer than broad, with a pair of well-developed eyes, length of eyes 0.09 mm, breadth 0.03 mm, carapace surface evenly and strongly granular. Median and posterior furrows prominent, regularly granular. Dorsal setae of carapace, borne on larger but relatively inconspicuous tubercles. With 100–101 denticuloclavate setae, including 8 on anterior margin and 11–12 on posterior margin. ***Coxae***: manducatory process with 4 setae (1 long apical, 2 rather short subapical setae, and 1 suboral seta at base of medial margin). Pedipalpal coxa with 10 (non-denticulate) + 7–8 (denticulate) setae, coxa I 14–16, II 15, III 18–19, IV with an anterolateral process and 30–36 setae. Coxal sac occupying only 1/2 of coxal length, atrium well developed (Fig. 1E). ***Chelicera*** (Fig. 1B): 1.85–1.91 × longer than broad, hand with 5 setae and 1 lyrifissure dorsally, movable finger with 1 submedial seta (1 specimen with 2 submedial setae) and 2–3 teeth (Fig. 1B). Galea with a short, broad stump on left chelicera (clearly broken). Serrula exterior with about 21–22 blades. Rallum with 3 blades, anterior 1 weakly denticulate distally. ***Pedipalp*** (Fig. 1F–H): all segments with well-developed granulations, except for chelal fingers, which are smooth; dorsal setae short and prominently denticuloclavate. Trochanter 1.92–1.95 ×, femur 6.55–6.57 ×, patella 5.29–5.33 × longer than broad. Femur 1.13–1.14 × longer than patella. Chela with pedicel 6.10–6.12 ×, hand with pedicel 3.06–3.08 × longer than broad; movable chelal finger 1.03–1.04 × longer than hand with pedicel length. Fixed finger with 64–66 small cusped teeth, movable finger with 63–65 teeth; venom apparatus present in both chelal fingers very short (Fig. 1G). Fixed chelal finger with 8 trichobothria and movable finger with 4, *eb*-*esb* (retrolateral view) and *ib-isb* (dorsal view) at the base of the fixed finger; *est* in finger middle, *et* distinctly closer to fingertip than to *it*; on movable finger, *st* nearer to *sb* than to *t.****Opisthosoma***: tergal chaetotaxy (I–XI): 10: 12: 13: 14: 16: 16: 14: 19: 19: 15: 12; sternal chaetotaxy (IV–XI): 2 × 2 + 9: 11: 12: 12: 10: 14: 10: 9; anal cone with 2 dorsal and 2 ventral setae. Tergite XI with 2 tactile setae. Anterior genital operculum with 73–74 setae and 2 lyrifissures; posterior operculum with 16–17 setae, 7–8 lyrifissures (Fig. 1I). Structure of male genitalia as illustrated (Fig. 1J); eversible sacs large; apodeme of eversible sac and lateral apodeme well developed. ***Legs*** (Figs 1C–D): Leg I: surface with weak scale-like sculpture, trochanter 1.24–1.25 ×, femur 1.76–1.78 × longer than deep and 0.51–0.53 × longer than patella; patella 4.21–4.24 ×, tibia 5.90–5.93 ×, tarsus 5.11–5.16 × longer than deep, subterminal seta simple, claws modified and asymmetrical, lateral claw shorter than mesal one (Fig. 1C). Leg IV: trochanter 1.94–1.95 ×, femoropatella 4.91–4.95 ×, tibia 9.20–9.23 × longer than deep and tarsus 8.00–8.05 × longer than deep. Arolia on legs I and IV shorter than claws (Fig. 1C, D).

**Adult female** (Fig. 5B). Mostly the same as the holotype.

***Carapace***: slightly longer than broad (1.12–1.13 ×), anterior margin with 6 setae, posterior margin with 12–13 setae. Well-developed paramedian impressions behind eyes as in male. ***Coxae***: pedipalpal coxa with 14 setae, coxa I 12–15, II 19–21, III 22–25, IV 33–38. ***Chelicera***: 1.98–2.01 × longer than broad, movable finger with 3 teeth. ***Pedipalp***: trochanter 2.07–2.09 × longer than broad, femur 6.46–6.48 × longer than broad, patella 5.11–5.15 × longer than broad, femur 1.12–1.13 × longer than patella. Chela with pedicel 5.53–5.56 × longer than broad, hand with pedicel 2.82–2.84 × longer than broad; movable finger 1.02–1.03 × longer than hand with pedicel length. ***Opisthosoma***: tergal chaetotaxy (I–XI): 11: 14: 13: 14: 16: 18: 18: 18: 18: 16: 9; sternal chaetotaxy (IV–XI): 2 × 1 + 9: 15: 12: 13: 11: 14: 14: 7; anal cone with 2 dorsal and 2 ventral setae. Anterior genital operculum with 31 setae (24 of them tubular) and 2 lyrifissures; posterior operculum with 12 setae, without lyrifissures (Fig. 1K). Sternites with 2 lateral cribriform plates.

##### Dimensions

**(length/width or, in the case of the legs, length/depth in mm). Males** (females in parentheses): body length 2.52–2.74 (3.70–3.92). Carapace 0.96–0.98/0.84–0.86 (1.05–1.09/0.94–0.96). Pedipalp: trochanter 0.50–0.52/0.26–0.27 (0.58–0.60/0.28–0.29), femur 1.44–1.46/0.22–0.23 (1.55–1.58/0.24–0.25), patella 1.27–1.29/0.24–0.25 (1.38–1.41/0.27–0.28), hand with pedicel 0.95–0.97/0.31–0.32 (1.07–1.10/0.38–0.39), length of movable chelal finger 0.98–0.99 (1.09–1.11), chela 1.89–1.93/0.31–0.32 (2.10–2.13/0.38–0.39). Chelicera: 0.24–0.26/0.13–0.14 (0.26–0.28/0.14–0.15). Leg I: trochanter 0.21–0.22/0.17–0.18 (0.25–0.26/0.18–0.19), femur 0.30–0.32/0.17–0.18 (0.35–0.37/0.17–0.18), patella 0.59–0.61/0.14–0.15 (0.61–0.65/0.15–0.16), tibia 0.59–0.60/0.10–0.11 (0.65–0.66/0.10–0.11), tarsus 0.46–0.49/0.09–0.10 (0.65–0.67/0.10–0.11). Leg IV: trochanter 0.33–0.34/0.17–0.18 (0.35–0.37/0.23–0.24), femoropatella 1.08–1.10/0.22–0.23 (1.06–1.09/0.25–0.26), tibia 0.92–0.95/0.10–0.11 (0.98–0.99/0.12–0.13), tarsus 0.64–0.65/0.08–0.09 (0.65–0.68/0.09–0.10).

##### Distribution.

Thailand (Nakhon Ratchasima).

#### ﻿ Metachelifer mahnerti sp. nov.

Taxon classificationAnimaliaPseudoscorpionesCheliferidae

112F1E3F-3E42-57AC-903C-8E101527A42C

http://zoobank.org/E787CC96-790B-4359-9632-8419F8AA5501

Figs 2 , 5C, D

##### Type material.

***Holotype*** male: Thailand, Chiangmai Province, Chom Thong district, Ban Luang Village, Tham Borichinda Cave, 18°29'53.01"N, 98°40'49.97"E, 379 m a.s.l., 15 Oct. 2014, Yun-Chun Li and Zhi-Gang Chen leg., in MCWNU (Ms20141015-01). ***Paratypes***: 1 male, 7 females, 1 tritonymph, collected with the holotype in MCWNU (Ms20141015-01); 1 male, 1 female, collected with the holotype, in MNHN.

##### Diagnosis.

Troglobiont habitus. This new species is distinguished from other members of the genus *Metachelifer* by the following combination of characters: anterior margin of carapace with 4 denticuloclavate setae and 91–93 setae; tergite XI without tactile setae; male anterior genital operculum with 68–70 setae (11–16 of them tubular); female anterior genital operculum without tubular setae, posterior operculum with 8 lyrifissures; and female body very large, 4.76–4.85 mm.

##### Etymology.

The new species is named in honour of the late Volker Mahnert (Muséum d’histoire naturelle, Genève, Switzerland).

##### Description.

**Adult male** (Fig. 5C). Carapace, pedipalps and tergites I–V dark brown, remaining parts yellowish brown (Fig. 5C).

**Figure 2.**
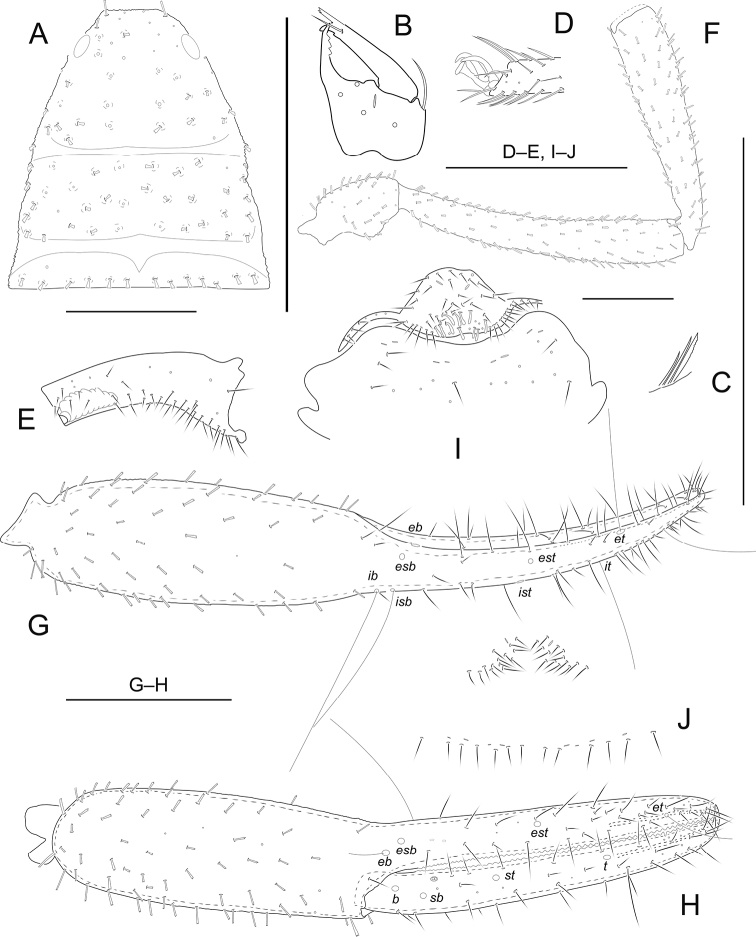
*Metachelifermahnerti* sp. nov., holotype male (**A–I**) and paratype female (**J**) **A** carapace **B** left chelicera **C** rallum of left chelicera **D** detail on tarsus I, lateral view **E** coxa IV, ventral view **F** palp (minus chela) **G** chela, retrolateral view **H** chela, dorsal view **I** male genital area **J** female genital area. Scale bars: 0.50 mm.

***Carapace*** (Fig. 2A): 1.05–1.06 × longer than broad, with a pair of well-developed eyes, length of eyes 0.10 mm, breadth 0.04 mm, carapace surface evenly and strongly granular. Median and posterior furrows prominent, regularly granular. Dorsal setae of carapace, borne on larger but relatively inconspicuous tubercles. With 91–93 denticuloclavate setae, including 4 on anterior margin and 13–14 on posterior margin. ***Coxae***: manducatory process with 4 setae (1 long apical, 2 rather short subapical setae, and 1 suboral seta at base of medial margin). Pedipalpal coxa with 11–12 (non-denticulate) + 4–5 (denticulate) setae, coxa I 20–22, II 19–21, III 21–23, IV with an anterolateral process and 43–47 setae. Coxal sac occupying only 2/5 of coxal length, atrium well developed (Fig. 2E). ***Chelicera*** (Fig. 2B, C): 1.66–1.69 × longer than broad, hand with 5 setae and 1 lyrifissure dorsally, movable finger with 1 submedial seta and 3 teeth (Fig. 2B). Galea with 3 short branches. Serrula exterior with about 19–20 blades. Rallum with 3 blades, anterior 1 weakly denticulate distally (Fig. 2C). ***Pedipalp*** (Fig. 2F–H): all segments with well-developed granulations, except for chelal fingers, which are smooth; dorsal setae short and prominently denticuloclavate. Trochanter 1.97–1.99 × longer than broad, femur 6.86–6.90 × longer than broad, patella 4.89–4.91 × longer than broad, femur 1.14–1.15 × longer than patella. Chela with pedicel 6.11–6.14 × longer than broad, hand with pedicel 2.97–2.99 × longer than broad; movable finger 1.06–1.07 × longer than hand with pedicel length. Fixed finger with 58–60 small cusped teeth, movable finger with 57–60 teeth; venom apparatus present in both chelal fingers, very short (Fig. 2H). Fixed chelal finger with 8 trichobothria and movable finger with 4, *eb*-*esb* (retrolateral view) and *ib-isb* (dorsal view) at the base of the fixed finger; *est* in finger middle, *et* distinctly closer to fingertip than to *it*; on movable finger, *st* nearer to *sb* than to *t.****Opisthosoma***: tergal chaetotaxy (I–XI): 14: 16: 16: 18: 20: 24: 23: 20: 20: 13: 14; sternal chaetotaxy (IV–XI): 2 × 1 + 10: 12: 14: 12: 14: 13: 15: 13; anal cone with 2 dorsal and 2 ventral setae. Tergite XI without tactile setae. Because only two specimens were available for the study, the structure of the genitalia could not be examined in detail. Anterior genital operculum with 68–70 setae (11–16 of them tubular) and 2 lyrifissures; posterior operculum with 17–19 setae, 8 lyrifissures (Fig. 2I). ***Legs***: Leg I: surface with weak scale-like sculpture, trochanter 1.35–1.36 ×, femur 1.84–1.86 × longer than deep and 0.58–0.60 × longer than patella; patella 4.00–4.02 ×, tibia 5.00–5.03 ×, tarsus 6.11–6.14 × longer than deep, subterminal seta simple, claws modified and asymmetrical, lateral claw shorter than mesal one (Fig. 2D). Leg IV: trochanter 1.63–1. 64 × longer than deep, femoropatella 3.93–3.96 ×, tibia 7.38–7.40 × longer than deep and tarsus 7.22–7.26 × longer than deep. Arolia on legs I and IV shorter than claws.

**Adult female** (Fig. 5 D). Mostly the same as the holotype.

***Carapace***: slightly longer than broad (1.00–1.01 ×), anterior margin with 4 setae, posterior margin with 12–13 setae. Well-developed paramedian impressions behind eyes like in male. ***Coxae***: pedipalpal coxa with 9 (non-denticulate) + 4–6 (denticulate) setae, coxa I 20–21, II 18, III 17–19, IV 52–55. ***Chelicera***: 1.94–1.97 × longer than broad, movable finger with 2–3 teeth. ***Pedipalp***: trochanter 2.03–2.05 × longer than broad, femur 6.18–6.21 × longer than broad, patella 4.81–4.86 × longer than broad, femur 1.16–1.17 × longer than patella. Chela with pedicel 5.10–5.11 × longer than broad, hand with pedicel 2.51–2.53 × longer than broad; movable finger 1.01–1.02 × longer than hand with pedicel length. ***Opisthosoma***: tergal chaetotaxy (I–XI): 15: 18: 20: 24: 23: 25: 25: 26: 23: 16: 15; sternal chaetotaxy (IV–XI): 2 × 1 + 11: 15: 15: 15: 13: 14: 13: 10; anal cone with 2 dorsal and 2 ventral setae. Anterior genital operculum with 28 setae (without tubular setae) and 2 lyrifissures; posterior operculum with 13 setae, 8 lyrifissures (Fig. 2J). Sternites with 2 lateral cribriform plates.

##### Dimensions

**(length/width or, in the case of the legs, length/depth in mm). Males** (females in parentheses): body length 3.23–3.35 (4.76–4.85). Carapace 1.05–1.06/1.00–1.01 (1.21–1.23/1.21–1.22). Pedipalp: trochanter 0.59–0.61/0.30–0.31 (0.67–0.69/0.33–0.35), femur 1.51–1.53/0.22–0.24 (1.73–1.75/0.28–0.29), patella 1.32–1.34/0.27–0.29 (1.49–1.51/0.31–0.33), hand with pedicel 1.04–1.05/0.35–0.37 (1.23–1.25/0.49–0.51), length of movable chelal finger 1.10–1.11 (1.24–1.26), length of chela 2.14–2.15/0.35–0.37 (2.50–2.53/0.49–0.51). Chelicera: 0.26–0.27/0.14–0.15 (0.28–0.30/0.14–0.15). Leg I: trochanter 0.23–0.25/0.17–0.18 (0.31–0.33/0.22–0.23), femur 0.35–0.37/0.19–0.20 (0.30–0.32/0.21–0.23), patella 0.60–0.61/0.15–0.16 (0.76–0.79/0.16–0.17), tibia 0.60–0.62/0.12–0.13 (0.73–0.75/0.11–0.12), tarsus 0.55–0.56/0.09–0.10 (0.70–0.71/0.10–0.11). Leg IV: trochanter 0.31–0.33/0.19–0.21 (0.39–0.41/0.25–0.26), femoropatella 1.06–1.08/0.27–0.28 (1.18–1.20/0.32–0.33), tibia 0.96–0.97/0.13–0.14 (1.15–1.16/0.14–0.15), tarsus 0.65–0.67/0.09–0.10 (0.78–0.80/0.10–0.11).

##### Distribution.

Thailand (Chiangmai).

#### ﻿ Metachelifer takensis sp. nov.

Taxon classificationAnimaliaPseudoscorpionesCheliferidae

CA24B6DD-A8D6-5EB1-9BC0-9D516A22EB45

http://zoobank.org/111C7F85-255E-4A4B-9C29-F9AAAA4AC6ED

Figs 3 , 6A, B

##### Type material.

***Holotype*** male: Thailand, Tak Province, Umphang district, Umphang subdistrict, Huai Lao Poo Cave, 15°57.680'N, 098°52.510'E, 534 m a.s.l., 16 Nov 2016, Yun-Chun Li and Zhi-Gang Chen leg, in MCWNU (Ms20161116-01). ***Paratypes***: 1 male, 1 female, 4 tritonymphs, collected with the holotype, in MCWNU (Ms20161116-01).

##### Diagnosis.

Troglobiont habitus. This new species is distinguished from other members of the genus *Metachelifer* by the following combination of characters: coxa IV with 45–50 setae; movable finger with 2 pseudotactile setae; male anterior genital operculum with 75–80 setae (without tubular setae); female anterior genital operculum with 22 setae (without tubular setae), posterior operculum with 8 lyrifissures.

##### Etymology.

Latinized adjective, derived from the province of Tak, where the type locality is located.

##### Description.

**Adult male** (Fig. 6A). Carapace, pedipalps and tergites dark brown, remaining parts yellowish brown (Fig. 6 A).

**Figure 3.**
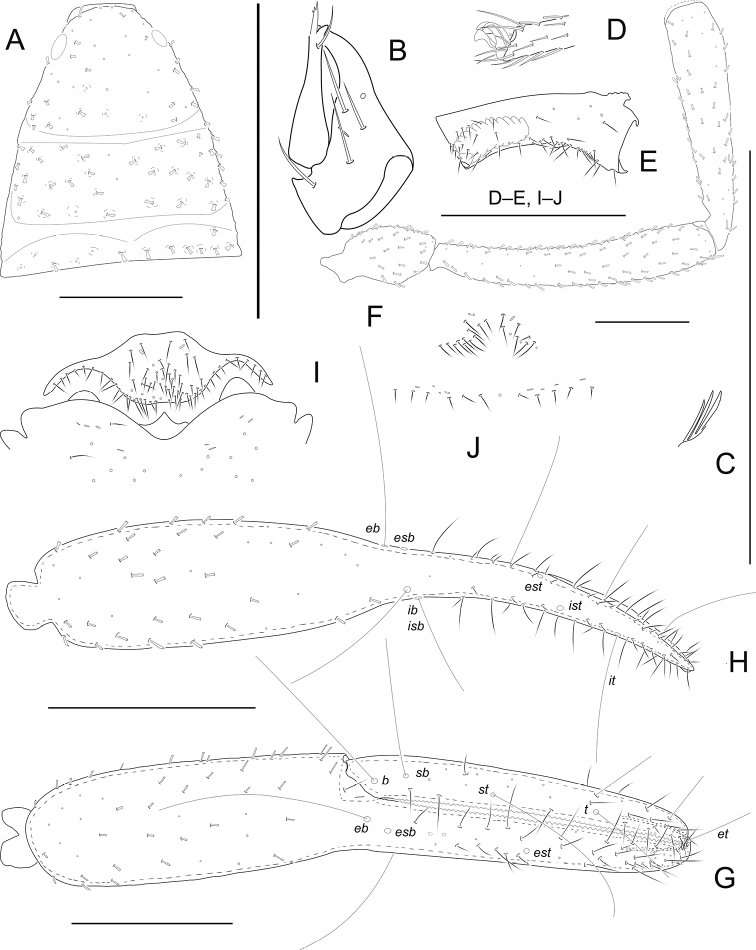
*Metachelifertakensis* sp. nov., holotype male (**A–I**) and paratype female (**J**) **A** carapace **B** left chelicera **C** rallum of left chelicera **D** detail on tarsus I, lateral view **E** coxa IV, ventral view **F** palp (minus chela) **G** chela, retrolateral view **H** chela, dorsal view **I** male genital area **J** female genital area. Scale bars: 0.50 mm.

***Carapace*** (Fig. 3A): 1.06–1.08 × longer than broad, with a pair of well-developed eyes, length of eyes 0.11 mm, breadth 0.05 mm, carapace surface evenly and strongly granular. Median and posterior furrows prominent, regularly granular. Dorsal setae of carapace, borne on larger but relatively inconspicuous tubercles. With 95–96 denticuloclavate setae, including 6 on anterior margin and 11–12 on posterior margin. ***Coxae***: manducatory process with a total of 4 setae (1 long apical, 1 rather short subapical seta, and 2 suboral setae at base of medial margin). Pedipalpal coxa with 11–12 (non-denticulate) + 6–7 (denticulate) setae, coxa I 13–15, II 15–17, III 15–19, IV with an anterolateral process and 45–50 setae. Coxal sac occupying only 2/5 of coxal length, atrium well developed (Fig. 3E). ***Chelicera*** (Fig. 3B, C): 1.75–1.80 × longer than broad, hand with 5 setae and 1 lyrifissure dorsally, movable finger with 1 submedial seta and 1–2 teeth (Fig. 3B). Galea with 3 short branches (Fig. 3B). Serrula exterior with about 17–18 blades. Rallum with 3 blades, anterior one weakly denticulate distally (Fig. 3C). ***Pedipalp*** (Figs 3F–H): all segments with well-developed granulation, except for chelal fingers, which are smooth; dorsal setae short and prominently denticuloclavate. Trochanter 2.00–2.01 × longer than broad, femur 6.04–6.06 × longer than broad, patella 4.64–4.65 × longer than broad, femur 1.16–1.17 × longer than patella. Chela with pedicel 5.94–5.97 × longer than broad, hand with pedicel 2.97–2.99 × longer than broad; movable finger 1.02–1.03 × longer than hand with pedicel length. Fixed finger with 61–62 small cusped teeth, movable finger with 62 teeth; venom apparatus present in both chelal fingers, very short (Fig. 3G). Fixed chelal finger with 8 trichobothria and movable finger with 4, *eb*-*esb* (retrolateral view) and *ib-isb* (dorsal view) at the base of the fixed finger; *est* in finger middle, *et* distinctly closer to fingertip than to *it*; on movable finger, with two pseudotactile setae, one nearer fingertip, one nearer *t* and on same level, *st* nearer to *sb* than to *t.****Opisthosoma***: tergal chaetotaxy (I–XI): 12: 15: 13: 14: 13: 21: 16: 18: 15: 14: 13; sternal chaetotaxy (IV–XI): 2 × 1 + 9: 12: 14: 13: 12: 11: 8: 11; anal cone with 2 dorsal and 2 ventral setae. Tergite XI with 2 tactile setae. Because only two specimens were available for the study, the structure of the genitalia could not be examined in detail. It was only possible to see well visible eversible sacs (ramshorn organs). Anterior genital operculum with 75–80 setae (without tubular setae) and 2 lyrifissures; posterior operculum with 16 setae, 8 lyrifissures (Fig. 3I). ***Legs***: Leg I: surface with weak scale-like sculpture, trochanter 1.39–1.40 ×, femur 1.53–1.55 × longer than deep and 0.43–0.45 × longer than patella; patella 4.29–4.32 ×, tibia 4.75–4.77 ×, tarsus 5.67–5.70 × longer than deep, subterminal seta simple, claws modified and asymmetrical, lateral claw shorter than mesal one (Fig. 3D). Leg IV: trochanter 1.77–1.79 ×, femoropatella 3.39–3.41 ×, tibia 6.92–6.95 × longer than deep and tarsus 6.56–6.58 × longer than deep. Arolia on legs I and IV shorter than claws (Fig. 3D).

**Adult female** (Fig. 6B). Mostly the same as the holotype.

***Carapace***: slightly longer than broad (1.10 ×), anterior margin with 4 setae, posterior margin with 10 setae. Well-developed paramedian impressions behind eyes like in male. ***Coxae***: pedipalpal coxa with 14 (non-denticulate) + 4 (denticulate) setae, coxa I 11, II 15, III 23, IV 46. ***Chelicera***: 1.79 × longer than broad, movable finger with 2 teeth. ***Pedipalp***: trochanter 1.88 × longer than broad, femur 6.04 × longer than broad, patella 4.37 × longer than broad, femur 1.15 × longer than patella. Chela with pedicel 5.31 × longer than broad, hand with pedicel 2.71 × longer than broad; movable finger 1.01 × longer than hand with pedicel length. ***Opisthosoma***: tergal chaetotaxy (I–XI): 14: 16: 14: 17: 20: 18: 19: 20: 18: 14: 13; sternal chaetotaxy (IV–XI): 2 × 1 + 11: 13: 14:14: 13: 12: 10: 10; anal cone with 2 dorsal and 2 ventral setae. Anterior genital operculum with 22 setae (without tubular setae) and 2 lyrifissures; posterior operculum with 12 setae, 8 lyrifissures (Fig. 3J). Sternites with 2 lateral cribriform plates.

##### Dimensions

**(length/width or, in the case of the legs, length/depth in mm). Males** (female in parentheses): body length 3.63–3.75 (3.52). Carapace 1.05–1.06/0.99–1.00 (1.11/1.01). Pedipalp: trochanter 0.60–0.62/0.30–0.31 (0.60/0.32), femur 1.51–1.53/0.25–0.26 (1.51/0.25), patella 1.30–1.32/0.28–0.29 (1.31/0.30), hand with pedicel 1.07–1.09/0.36–0.38 (1.14/0.42), length of movable chelal finger 1.09–1.10 (1.15), length of chela 2.14–2.17/0.36–0.38 (2.23/0.42). Chelicera: 0.32–0.34/0.19–0.20 (0.25/0.14). Leg I: trochanter 0.25–0.26/0.18–0.19 (0.25/0.19), femur 0.26–0.28/0.17–0.18 (0.35/0.18), patella 0.60–0.62/0.14–0.15 (0.62/0.15), tibia 0.57–0.59/0.12–0.13 (0.62/0.15), tarsus 0.51–0.52/0.09–0.10 (0.53/0.08). Leg IV: trochanter 0.39–0.41/0.22–0.23 (0.42/0.21), femoropatella 0.95–0.97/0.28–0.29 (1.05/0.29), tibia 0.90–0.92/0.13–0.14 (0.92/0.13), tarsus 0.59–0.60/0.09–0.10 (0.61/0.09).

##### Distribution.

Thailand (Tak).

#### ﻿ Metachelifer thailandicus sp. nov.

Taxon classificationAnimaliaPseudoscorpionesCheliferidae

40D47B5E-2460-598C-B31F-4A053AC293DE

http://zoobank.org/5DD71EBC-2ECC-4EF9-9DF3-AA9D1E9045DD

Figs 4 , 6C, D

##### Type material.

***Holotype*** male: Thailand, Tak Province, Phop Phra district, Mae Ku subdistrict, Tham Sua Yai Cave, 16°40.336'N, 98°40.138'E, 466 m a.s.l., 14 Nov 2016, Yun-Chun Li and Zhi-Gang Chen leg., in MCWNU (Ms20161116-01). ***Paratypes***: 1 male, 4 females, collected with the holotype in MCWNU (Ms20161116-01).

##### Diagnosis.

Troglobiont habitus. This new species is distinguished from other members of the genus *Metachelifer* by the following combination of characters: anterior margin of carapace with 6 denticuloclavate setae and a total of 86–88 setae; chelicera galea with 2 short branches; male movable chelal finger 0.96–0.98 × and female 0.93–0.95 × longer than hand with pedicel length; male anterior genital operculum without tubular setae; female genital posterior operculum with 6 setae, without lyrifissures.

##### Etymology.

Latinized adjective, derived from the country of Thailand, where the type locality is located.

##### Description.

**Adult male** (Fig. 6C). Carapace and pedipalps dark brown, remaining parts yellowish brown (Fig. 6C).

**Figure 4.**
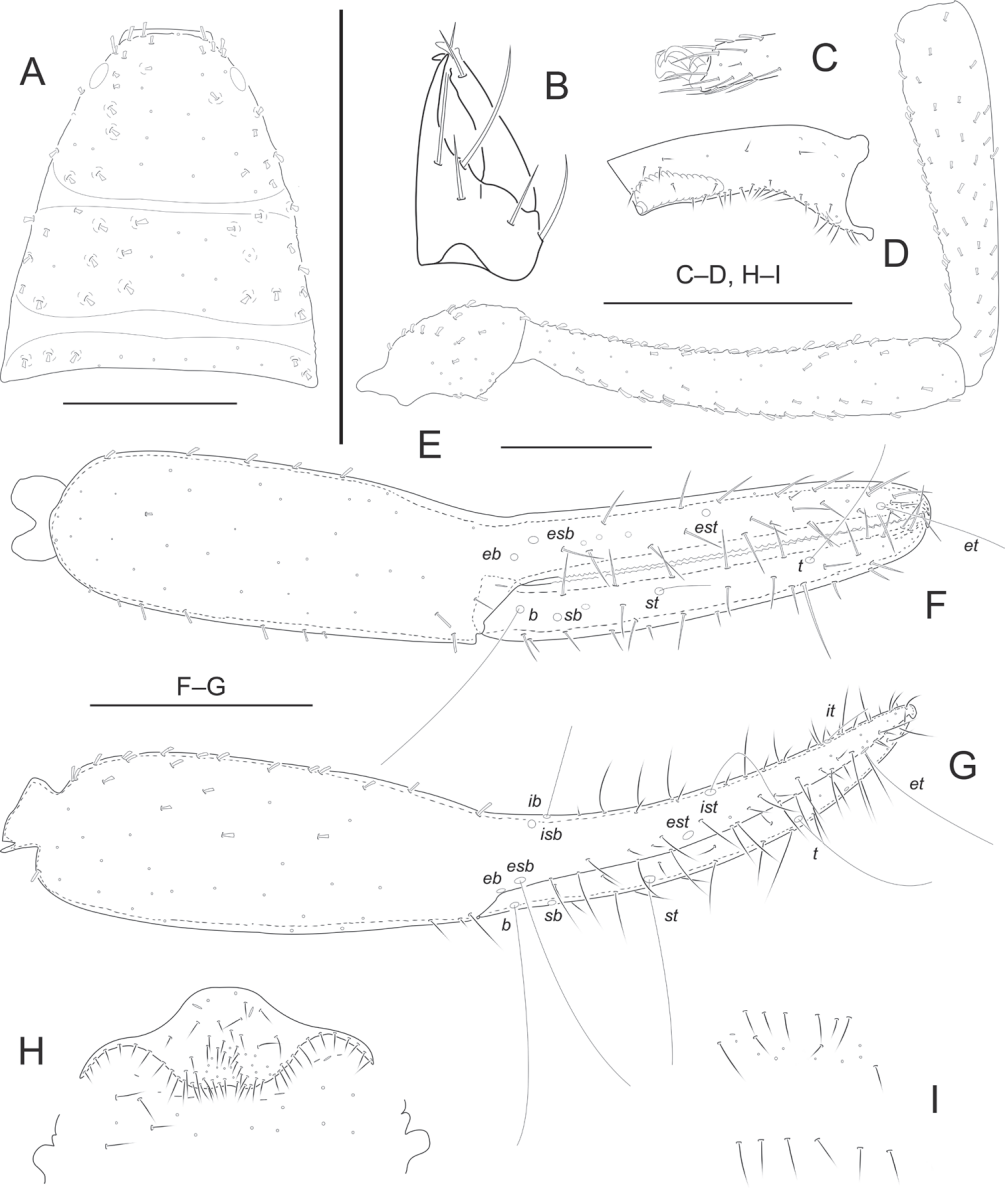
*Metacheliferthailandicus* sp. nov., holotype male (**A–H**) and paratype female (**I**) **A** carapace **B** right chelicera **C** detail on tarsus I, lateral view **D** coxa IV, ventral view **E** palp (minus chela) **F** chela, retrolateral view **G** chela, dorsal view **H** male genital area **I** female genital area. Scale bars: 0.50 mm.

***Carapace*** (Fig. 4A): 1.06–1.07 × longer than broad, with a pair of well-developed eyes, length of eyes 0.10 mm, breadth 0.04 mm, carapace surface evenly and strongly granular. Median and posterior furrows prominent, regularly granular. Dorsal setae of carapace, borne on larger but relatively inconspicuous tubercles. With a total of 86–88 denticuloclavate setae, including 6 on anterior margin and 12–13 on posterior margin. ***Coxae***: manducatory process with 4 setae (1 long apical, 1 rather short subapical seta, and 2 suboral setae at base of medial margin). Pedipalpal coxa with 11–12 (non-denticulate) + 7–8 (denticulate) setae, coxa I 9–11, II 13–15, III 15–18, IV with an anterolateral process and 39–45 setae. Coxal sac occupying only 2/5 of coxal length, atrium well developed (Fig. 4D). ***Chelicera*** (Fig. 4B): 1.90–1.91 × longer than broad, hand with 5 setae and 1 lyrifissure dorsally, movable finger with 1 submedial seta and 1–2 teeth (Fig. 4B). Galea with 2 short branches. Serrula exterior with about 18–20 blades. Rallum with 3 blades, anterior one weakly denticulate distally. ***Pedipalp*** (Figs 4E–G): all segments with well-developed granulations, except for chelal fingers, which are smooth; dorsal setae short and prominently denticuloclavate. Trochanter 2.00–2.03 × longer than broad, femur 6.13–6.15 × longer than broad, patella 4.74–4.77 × longer than broad, femur 1.15–1.16 × longer than patella. Chela with pedicel 5.50–5.53 × longer than broad, hand with pedicel 2.84–2.85 × longer than broad; movable finger 0.96–0.98 × longer than hand with pedicel length. Fixed finger with 59–61 small cusped teeth, movable finger with 58–61 teeth; venom apparatus present in both chelal fingers, very short (Fig. 4F). Fixed chelal finger with 8 trichobothria and movable finger with 4, *eb*-*esb* (retrolateral view) and *ib-isb* (dorsal view) at the base of the fixed finger; *est* in finger middle, *et* distinctly closer to fingertip than to *it*; on movable finger, with one pseudotactile seta, nearer *t*, but the latter distinctly closer to fingertip, *st* nearer to *sb* than to *t.****Opisthosoma***: tergal chaetotaxy (I–XI): 14: 13: 13: 17: 14: 15: 18: 13: 13: 13: 9; sternal chaetotaxy (IV–XI): 2 × 1 + 11: 12: 11: 10: 9: 11: 12: 9; anal cone with 2 dorsal and 2 ventral setae. Tergite XI with 2 tactile setae. Because only two specimens were available for the study, the structure of the genitalia could not be examined in detail. Anterior genital operculum with 74–78 setae (without tubular setae) and 2 lyrifissures; posterior operculum with 14–15 setae, 8–9 lyrifissures (Fig. 4H). ***Legs***: Leg I: surface weakly scale-like sculptured, trochanter 1.33–1.36 ×, femur 2.06–2.08 × longer than deep and 0.63–0.64 × longer than patella; patella 4.00–4.03 ×, tibia 4.67–4.69 ×, tarsus 5.00–5.02 × longer than deep, subterminal seta simple, claws modified and asymmetrical, lateral claw shorter than mesal one (Fig. 4C). Leg IV: trochanter 1.94–1.95 ×, femoropatella 3.36–3.39 ×, tibia 7.50–7.53 × longer than deep and tarsus 6.67–6.71 × longer than deep. Arolia on legs I and IV shorter than claws (Fig. 4C).

**Adult female** (Fig. 6D). Mostly the same as the holotype. ***Carapace***: Slightly longer than broad (1.01–1.02 ×), posterior margin with 8–9 setae. Well-developed paramedian impressions behind eyes like in male. ***Coxae***: pedipalpal coxa with 11–13 (non-denticulate) + 5–6 (denticulate) setae, coxa I 11, II 15, III 23, IV 46. ***Chelicera***: 1.80–1.86 × longer than broad, movable finger with 2 teeth. ***Pedipalp***: trochanter 2.04–2.06 × longer than broad, femur 5.94–5.97 × longer than broad, patella 4.29–4.31 × longer than broad, femur 1.19–1.20 × longer than patella. Chela with pedicel 5.47–5.50 × longer than broad, hand with pedicel 2.87–2.89 × longer than broad; movable finger 0.93–0.95 × longer than hand with pedicel length. ***Opisthosoma***: tergal chaetotaxy (I–XII): 10: 9: 11: 9: 10: 12: 11: 11: 12: 11: 10; sternal chaetotaxy (IV–XII): 2 ×1 + 9: 12: 13: 14: 13: 12: 11: 10; anal cone with 2 dorsal and 2 ventral setae. Anterior genital operculum with 18–20 setae (without tubular setae) and 1 lyrifissure; posterior operculum with 6 setae, without lyrifissures (Fig. 4I). Sternites with 2 lateral cribriform plates.

**Figure 5.**
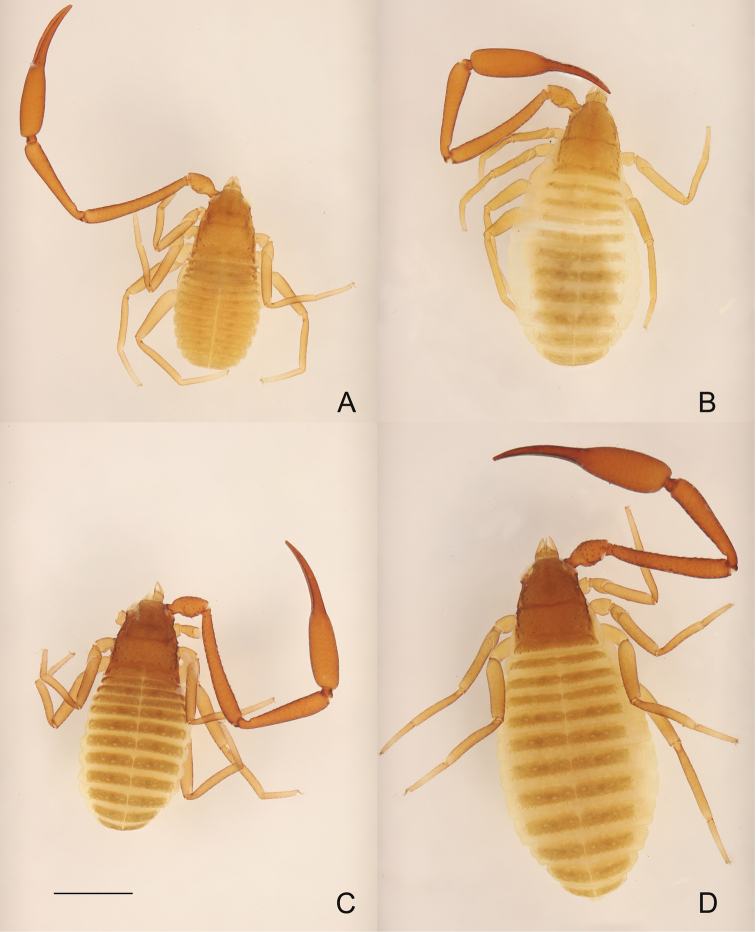
**A, B***Metachelifercheni* sp. nov., dorsal views **A** holotype male **B** paratype female **C, D***M.mahnerti* sp. nov., dorsal views **C** holotype male **D** paratype female. Scale bar: 1.00 mm (**A–D**).

**Figure 6.**
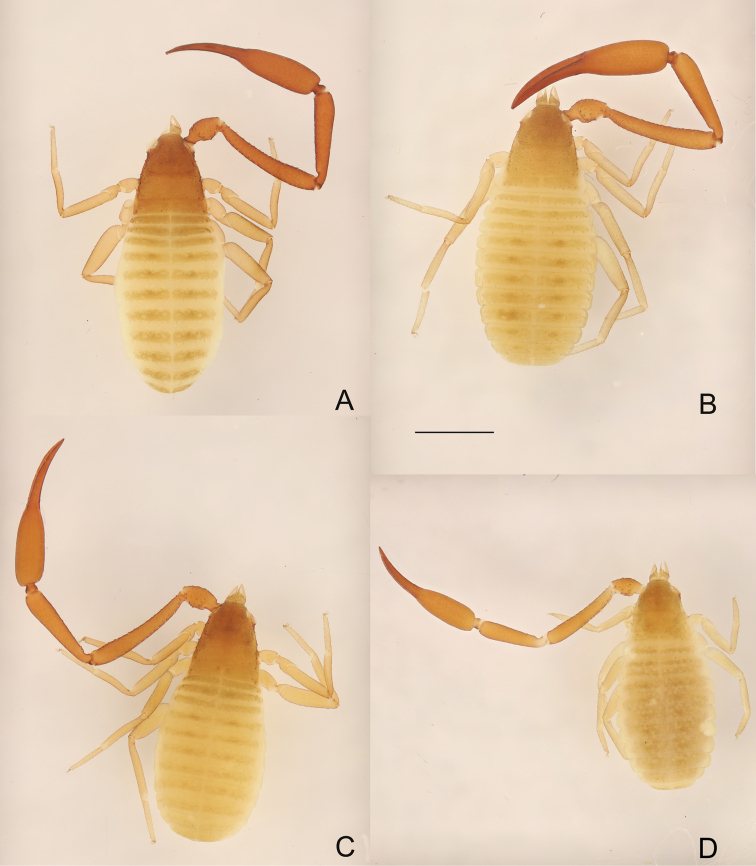
**A, B***Metachelifertakensis* sp. nov., dorsal views **A** holotype male **B** paratype female **C, D***M.thailandicus* sp. nov., dorsal views **C** holotype male **D** paratype female. Scale bar: 1.00 mm (**A–D**).

##### Dimensions

**(length/width or, in the case of the legs, length/depth in mm). Males** (females in parentheses): body length 3.38–3.42 (2.86–3.32). Carapace 0.99–1.01/0.93–0.94 (0.81–0.82/0.80–0.81). Pedipalp: trochanter 0.60–0.62/0.30–0.31 (0.49–0.51/0.24–0.26), femur 1.47–1.49/0.24–0.25 (1.07–1.09/0.18–0.20), patella 1.28–1.30/0.27–0.28 (0.90–0.92/0.21–0.22), hand with pedicel 1.08–1.10/0.38–0.39 (0.86–0.89/0.30–0.32), length of movable chelal finger 1.04–1.06 (0.80–0.83), length of chela 2.09–2.11/0.38–0.39 (1.64–1.67/0.30–0.32). Chelicera: 0.27–0.28/0.14–0.15 (0.23–0.25/0.13–0.14). Leg I: trochanter 0.24–0.25/0.18–0.19 (0.18–0.20/0.14–0.16), femur 0.35–0.37/0.17–0.19 (0.21–0.23/0.14–0.15), patella 0.56–0.58/0.14–0.15 (0.46–0.49/0.13–0.14), tibia 0.56–0.58/0.12–0.13 (0.40–0.43/0.10–0.11), tarsus 0.50–0.51/0.10–0.11 (0.41–0.42/0.09–0.10). Leg IV: trochanter 0.33–0.34/0.17–0.19 (0.31–0.33/0.17–0.19), femoropatella 0.94–0.97/0.28–0.29 (0.72–0.75/0.22–0.23), tibia 0.90–0.93/0.12–0.13 (0.65–0.68/0.11–0.12), tarsus 0.60–0.61/0.09–0.10 (0.45–0.49/0.08–0.09).

##### Distribution.

Thailand (Tak).

## ﻿Discussion

**Figure 7.**
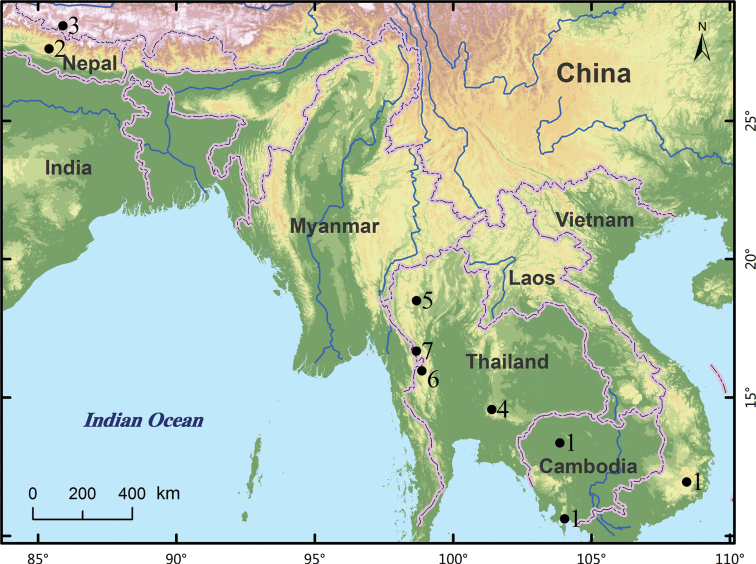
Distribution of known *Metachelifer* species. 1 *M.duboscqui*; 2 *M.macrotuberculatus*; 3 *M.nepalensis*; 4 *M.cheni* sp. nov.; 5 *M.mahnerti* sp. nov.; 6 *M.takensis* sp. nov.; 7 *M.thailandicus* sp. nov

Except for *Metachelifermacrotuberculatus* and *M.nepalensis* in Nepal, all other species of *Metachelifer* are distributed in Southeast Asia (Fig. 7). The new species described here all inhabit a low light area about 5–7 m from the entrance of the cave; they were collected from under a mixture of stones and large clods with a slightly drier surface. These species were not found in the environment around the cave entrance. In comparison with species living under tree bark, the length of male pedipalpal patella (tree-dwelling max. 1.20 mm vs cave-dwelling min. 1.27 mm), movable chelal finger (tree-dwelling max. 0.94 mm vs cave-dwelling min. 0.98 mm), pedal tibia I (tree-dwelling max 0.45 mm vs cave-dwelling min 0.56 mm), and pedal tarsus I (tree-dwelling max. 0.45 mm vs. cave-dwelling min. 0.46 mm) are much longer (Redikorzev 1938; Dashdamirov 2006), which suggests that these species are adapt to the cave environment.

## Supplementary Material

XML Treatment for Metachelifer

XML Treatment for Metachelifer cheni

XML Treatment for Metachelifer mahnerti

XML Treatment for Metachelifer takensis

XML Treatment for Metachelifer thailandicus
